# Rainfall-runoff modeling of urban floods using GIS and HEC-HMS

**DOI:** 10.1016/j.mex.2025.103437

**Published:** 2025-06-13

**Authors:** Priyanka Sharad Jawale, A.D. Thube

**Affiliations:** a College of Engineering Pune Technological University (Formerly College of Engineering Pune), Pune, India; b Dr. D. Y. Patil Institute of Engineering, Management and Research, Akurdi, Pune, India

**Keywords:** Urban runoff modelling using GIS and HEC-HMS, Modelling, Simulation, HEC-HMS, GIS

## Abstract

This research centres on simulating rainfall-runoff behaviour within the urban areas of Wakad Watershed in PCMC, Maharashtra, India, with a strong focus on improving water resource planning and availability assessments. Reliable forecasting of water availability is essential for sustainable water management, yet it often suffers from data limitations. To address this, the study employs advanced rainfall-runoff modelling techniques to offer accurate insights into the watershed’s hydrological processes, aiding data-driven decision-making. The watershed was meticulously delineated using a 7 m accuracy high-resolution Digital Elevation Model (DEM) coupled with 30 years of rainfall data from 1993 to 2023. GIS modelling in conjunction with HEC-HMS was carried out using the Soil Conservation Service Curve Number (SCS-CN), SCS Unit Hydrograph (SCS-UH), and Muskingum routing techniques. These approaches effectively integrated rainfall and watershed morphology details like slope, land use, soil properties, and climatic conditions. The model’s accuracy in predictions was enhanced through model calibration. The coefficient of determination (R^2^) exhibited 4.14 % increment improvement being raised from 0.67 to 0.699. NASH improved 44.23 % to a value of 0.624 displaying a competitive model. The Root Mean Square Error (RMSE) reduction of 51.90 % corroborated the effectiveness of the calibration made. RMSE fell from 1.235 to 0.813, while If PBIAS shifted from 43.69 % to 25.70 %. In summary, the model results indicate that the model demonstrates reasonable accuracy in representing the hydrological behaviour of the Wakad Watershed and provides a basis for developing water management plans in proactive environments of urban expansion.

• Possessing highly detailed (7 m) DEM, as well as long term (30 years) precipitation data, allowed high-accuracy flood modelling alongside precise watershed characterization for the Wakad Watershed.
• The combined application of GIS with the HEC-HMS modelling system, including SCS-CN, SCS-UH, and Muskingum methods, served as a robust framework for simulating urban floods and managing infrastructural development in rapidly evolving regions such as Wakad, PCMC.
• Model calibration led to more accurate simulations with the tuned model achieving 44.23 % more Nash-Sutcliffe Efficiency, 51.90 % less RMSE, 4.14 % increment in R^2^ and 41.2 % less Percent Bias while demonstrating effective simulation of rainfall-runoff processes.

## Graphical abstract

**Figure:**
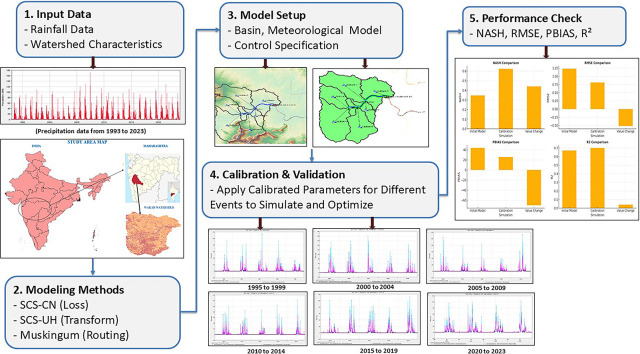

Specifications table

**Table:** 

Subject area	Engineering
More specific subject area	*Hydrology and Water Resources Engineering*
Name of your method	*Urban Runoff Modelling using GIS and HEC-HMS*
Name and reference of original method	*Not Applicable*
Resource availability	*The links of software used:* • *HEC-HMS*: *https://www.hec.usace.army.mil/software/hec-hms/*

## Introduction

The measurement of flood has gained much significance for areas in India specifically geographic regions in the recent past because of frequent occurrence of intense urban floods. From the numerous methodologies that are available the combination of GIS with hydrological models like HEC-HMS is one that is considered to be at the frontier in the assessment as well as in the management of floods. Suburbanization has caused major changes to the water cycle and water retention has become much less efficient, as natural ground has been replaced with cement and blacktop [1]. This change diminishes water penetration and steps up the amount of water flow across the surface which contributes to clogged drainage system and higher chances of flash floods, loss of property and even loss of life. The analysis with the help of HEC-HMS along with GIS implication offer a solid ground with quantification of rainfall-runoff system to predict the scenarios of future urban floods. By integrating the land use and cover data, rainfall intensity and characteristics of the drainage network the method offers accurate flood risk and supplements the strategies for disaster mitigation. Past flood control strategies using fixed structures and previous records and experience prove inadequate to handle flood operations in the current societies [2]. These models bear these limits when applied on the variability in rainfall and rising trends in urbanization. Consequently, failure to incorporate important factors such as changes in land use and the expansion of impervious surface area leads to poor flood estimates and hence poor measures of flood prevention. Unlike GIS and HEC-HMS, the capabilities of which are dynamic, enabling assessment in accordance with the current data, such as rainfall and land use change. These enhanced instruments allow the researchers and the planners to analyze the complexity of the flood occurrence in the urban areas with increased feasibility and accuracy. In addition, this type of methodology provides for the optimal solution with the trade-off between the level of accuracy and the time spent, and may be successfully used in facilities with limited resources. GIS and HEC-HMS tools are helpful approaches that could mitigate the shortcomings such as lack of data and computational difficulties to provide a robust model for predicts and manage the urban flood risk in future of India [3].

Techniques such as GIS coupled with sophisticated hydrological models have greatly enhanced the effectiveness in evaluating and maybe mitigating urban flood risks. GIS allows for the utilization of superior graphic interface to analyze spatial data for understanding of land use/ topographic interface affecting the runoff. When integrated with HEC-HMS, one of the most prominent hydrologic modelling tools, GIS improves the simulation of complex phenomena such as rainfall-runoff. HEC-HMS allows for flexibility in input data, allowing for simulations ranging from basic runoff to complicated floods including a reservoir, channel, and/or diversions. Another benefit of utilising HEC-HMS is the incorporation of the SCS-CN, SCS-UH, and Muskingum method approach to assist the calculation of runoff for % imperviousness, land use, slopes, and rainfall intensity [4]. Because of this approach, urban planners and disaster management teams are going to have a functional, yet precise way of evaluating flood risks. The process-based hydrologic models such as HEC-HMS plays an integrated role in such endeavours by mimicking precipitation and runoff patterns for the last several decades as well as help in the identification of flood prone areas to enable preventatively plan for improvements of drainage structures and construction of flood barriers. Due to the complexity in hydrological modeling, the Mula River Basin and Wakad Watershed located in pune India are chosen to study the applicability of GIS and HEC HMS in built up area flood modeling [5]. There is thus high level of urbanization in this region meaning that the surface area is mostly covered by concrete pavements etc resulting to altered hydrological characteristics of the region. Based on the same software, this research narrows down the precipitation analysis on the surface runoff and discharge by simulating thirty-year precipitation data. The SCS-CN, SCS-UH, and Muskingum methods integration assists in computing the possibility between infiltration and runoff so that it assists in giving some analysis on the possibility of the watershed. This comprehensive embrace hilly areas to assess critical areas likely to flood and knowledge to determine effective measures to follow during construction. The results obtained with the model’s aid should then be checked against real-world outcomes for model output credibility, allowing officials to devise measures that promote improved levels of flood resistance. Due to the use of these tools, the study helps improve flood management in Pune and presents a reference point in the management of similar problems in other urban centers around the globe [6].

This paper discusses the challenges of GIS and HEC-HMS for the rainfall-runoff modeling of urban floods. The first concerns are the lack and the quality of higher resolution spatial and temporal data. Precise simulation is defined by DEMs, land use, and soil type and quantity and quality of rainfall, which most of the time are missing or years behind. The urban regions also under go frequent changes in land uses and occupants, hence, it is a challenge to capture current data [7]. A second major issue arises from the fact that since there is scarce flood data available for urban watersheds, the HEC-HMS model requires calibration and validation. Connection of GIS with HEC HMS requires technical knowledge and computational resources and is always time-consuming; it takes more time when there are many data sets. However, the problem description of the urban areas still faces the issues of complicated drainage networks and localized flood behavior, which could be masked in the model. Overcoming these challenges and difficulties remains crucial for obtaining the desired outcomes for modelling urban floods [8].

The growth of cities changes natural water flow processes in big ways by making areas like concrete and asphalt, which reduce water infiltration and enhance surface runoff. This leads to higher peak flows and increased flood risks in urban watersheds. Rapid urban growth changes land use patterns, causing the watershed’s natural water retention capacity to diminish and the drainage system to become overwhelmed during heavy rainfall events. The altered rainfall-runoff relationship intensifies flood frequency and severity, complicating flood management efforts. Understanding these hydrological impacts through advanced modeling techniques like GIS and HEC-HMS is crucial for analyzing urban flood dynamics in rapidly developing regions.

The current research centers its analysis on the use of GIS and HEC-HMS to simulate the rainfall-runoff processes in the Wakad Watershed and Mula River Basin. The research plan, therefore, seeks to integrate spatial analysis with hydraulic modeling to fill essential knowledge gaps of urban flood. Key objectives include [9]:

1. Applying GIS to investigate the effects of vicious urbanization on hydrological processes with special reference to change in the percentage imperviousness and generation of surface runoff.
2. Using HEC-HMS to simulate the relations between precipitation amounts and runoff as well as to determine the discharge rates over a longer period of time.
3. Updating existing climate data to improve the flood hazard prediction and planning for managing future flood risks.
4. The ability to ‘get it right’ time after time is underwritten by a robust validation process that compares simulation results to data and actual measurements.

It also examines the possibility of enhancing the rainfall-runoff model forecasts with modern approaches like the machine learning approach and real-time data assimilation technique. In doing this, this research fills the gaps of the previous works and develops quite novel solutions to enhance the design of sustainable and cyclone-resistant urban structures for the target areas [10].

## Research methods

### Study area

The Wakad Watershed is a vital hydrological sub-basin located within the Pimpri-Chinchwad Municipal Corporation (PCMC) area of Pune, Maharashtra. It is situated along the northern banks of the Mula River, which originates from the Mulshi Dam in the Western Ghats and flows eastward through the Pune. Due to its proximity to the Mula River and rapid urban development, Wakad is susceptible to flooding, especially during monsoon season. Understanding the hydrological dynamics and urban development patterns of the Wakad Watershed is crucial for effective water resource management, flood mitigation, and sustainable urban planning in the region.

The following geographic coordinates define the study area:

### Research tools and materials

A Digital Elevation Model (DEM), measures of rainfall and flow, and a set of land use and soil type maps made up the secondary data [11]. The secondary info came from publications and organisations that were relevant. The combination of the existing data and application of these advanced tools allows for enhanced prediction and understanding of urban flood processes in the Wakad area This study combines sophisticated geospatial techniques and hydrological modelling techniques including GIS and HEC-HMS to enable flood simulation and prediction of urban floods. The methodology combines high resolution spatial datasets, and principled hydrological modelling.

### Method details

Utilizing state-of-the-art geospatial technologies and hydrological modeling methods such as GIS and HEC-HMS, this study investigates and predicts urban floods. The approach is based on the integration of high-resolution spatial datasets and sound hydrology modeling practices. The effective use of secondary data, such as 7 m resolution Digital Elevation Model (DEM) and 30 years of precipitation records, forms the backbone of the study. These datasets are useful for understanding the basic properties of watersheds, amount of rainfall, water flow etc. Software such as QGIS were used to produce a SCS Curve Number (CN) Map that combines the land use, soil type and hydrologic condition that determines the likelihood of runoff [12]. Traditional approaches may employ less numerous premises and coarse data, which made computed runoff values less accurate. Many conventional practices also did not incorporate GIS tools into their broad mix of advanced visualization instruments and data analysis. Unlike the current study that adopts the HEC-HMS software which takes topographic, hydrologic, and climatic data to create runoff hydrographs and flood levels with similar accuracy. The SCS-CN, SCS-UH, and Muskingum Routing methods upgrade the model with the estimation of runoff according to the characteristics of soils and types of terrain, what can be used as a useful tool for the investigation of floods in large cities. This integrated approach therefore represents a step forward than previous methods since it offers point, areal estimates of the hydrological processes in question. The integration of high-resolution data and accurate modeling positively influences the possibilities of satisfactory flood prediction and efficient management of water resources; hence employing recent geographical tools is crucial for managing urban floods [13].

The model development structure utilising HEC-HMS is made up of five components using a variety of methodologies. Table 1 shows the methodology used in this investigation.

**Table 1:** Methods used for study.

Model	Method
Precipitation	Specified Hyetograph
Runoff Volume Models	SCS CN
Direct Runoff Models	SCH UH
Reach Routing Models	Muskingum Method

#### HEC HMS model development

The use of Geographic Information System (GIS) tools combined with the Hydrologic Engineering Center's Hydrologic Modelling System (HEC-HMS) greatly improves urban flood modelling by using essential spatial information. Tools like QGIS and HEC-GeoHMS allow us to establish watershed boundaries, elevations, and drainage networks in order to simulate water flows in cities. GIS tools provide Digital Elevation Models (DEM's) which allow us to source the topographic models [14]. DEM's show detailed representations of available elevation that allows us to calculate the slope of a watershed, flow direction and other watershed features. Therefore, using HEC-HMS with GIS does allow us to model water flowing over part of an urban watershed and its relationship to rainfall. GIS data allows us to take advantage of the SCS Curve Number (CN). The SCS CN method allows the develop of CN maps that the hydrologic engineer uses to classify areas based on land use, soil type and hydrology. The scripts in HEC-GeoHMS use this data to estimate the potential runoff for watersheds and help evaluate flood risk in different urban areas. The advantage or benefit of using HEC-HMS and GIS is we can reasonably model the flooding events with regards to the spatial conditions found in our urban environments. The urban areas heterogeneity such as different soil types, land uses, drainage systems and various other criteria we calibrated and verified our modelling using relative accurate observed runoff data to evaluate or compare our models’ simulated outputs Table 2, Table 3, Table 4.

**Table 2:** Parameters used in HEC HMS model.

Element	Component	Method	Parameter
Sub-basin	Surface	Simple Surface	Initial Storage Max Storage
	Loss	SCS CN	Initial Abstraction Curve Number Impervious
	Transform	SCS UH	Time Lag SCS
Reach	Reach Routing Method	Muskingum	Muskingum K

**Table 3:** SCS CN values for each subbasins in Wakad watershed.

name	centroid_x	centroid_y	area_sqkm	latitude	longitude	basinid	_count	_sum	_mean
Subbasin-1	366368.4	2057137	10.83478	18.60087	73.73341	1	133198	10844651	81.417521
Subbasin-2	365219.4	2055556	6.415325	18.58651	73.72263	2	78847	6292828	79.810621
Subbasin-3	365265.5	2052130	14.17619	18.55556	73.7233	3	174671	13115628	75.08761
Subbasin-4	367139.1	2054109	1.60965	18.57356	73.74092	4	19859	1568862	79.00005
Subbasin-5	368041.9	2051411	9.42025	18.54923	73.74965	5	116120	8763298	75.467603
Subbasin-6	368733	2053749	3.538878	18.57041	73.75604	6	43636	3539971	81.125012
Subbasin-7	369710.7	2056275	9.820433	18.59329	73.76514	7	121109	9944767	82.114186

**Table 4:** Lag time for each subbasin in standard graph type.

Subbasin	Graph Type	Lag Time (MIN)
Subbasin-1	Standard	120.3935405
Subbasin-2	Standard	91.811
Subbasin-3	Standard	120.31015
Subbasin-4	Standard	39.313
Subbasin-5	Standard	77.304
Subbasin-6	Standard	77.060
Subbasin-7	Standard	82.378

### Basin model processing

Fig. 4. This is the Basin Model of the Wakad Basin. It is a key part of using HEC-HMS to model rainfall and flow that causes flooding in cities [15]. Seven sub-basins make up the watershed, which is shown in the model. The components that constitute a watershed system include sub-basins, channels (reaches), reservoirs, junctions, diversions, sources, and sinks. For the truer picture of the watershed's hydrological features and flow behaviours at the time of event, the sub-basins are used. With the incorporation of the seven separate sub-basins the model has made it possible to simulate flow and runoff behaviors across more defined catchment areas; thus, giving a more detailed understanding of flood risk.

## Model setup and calibration

**Fig. 1:**
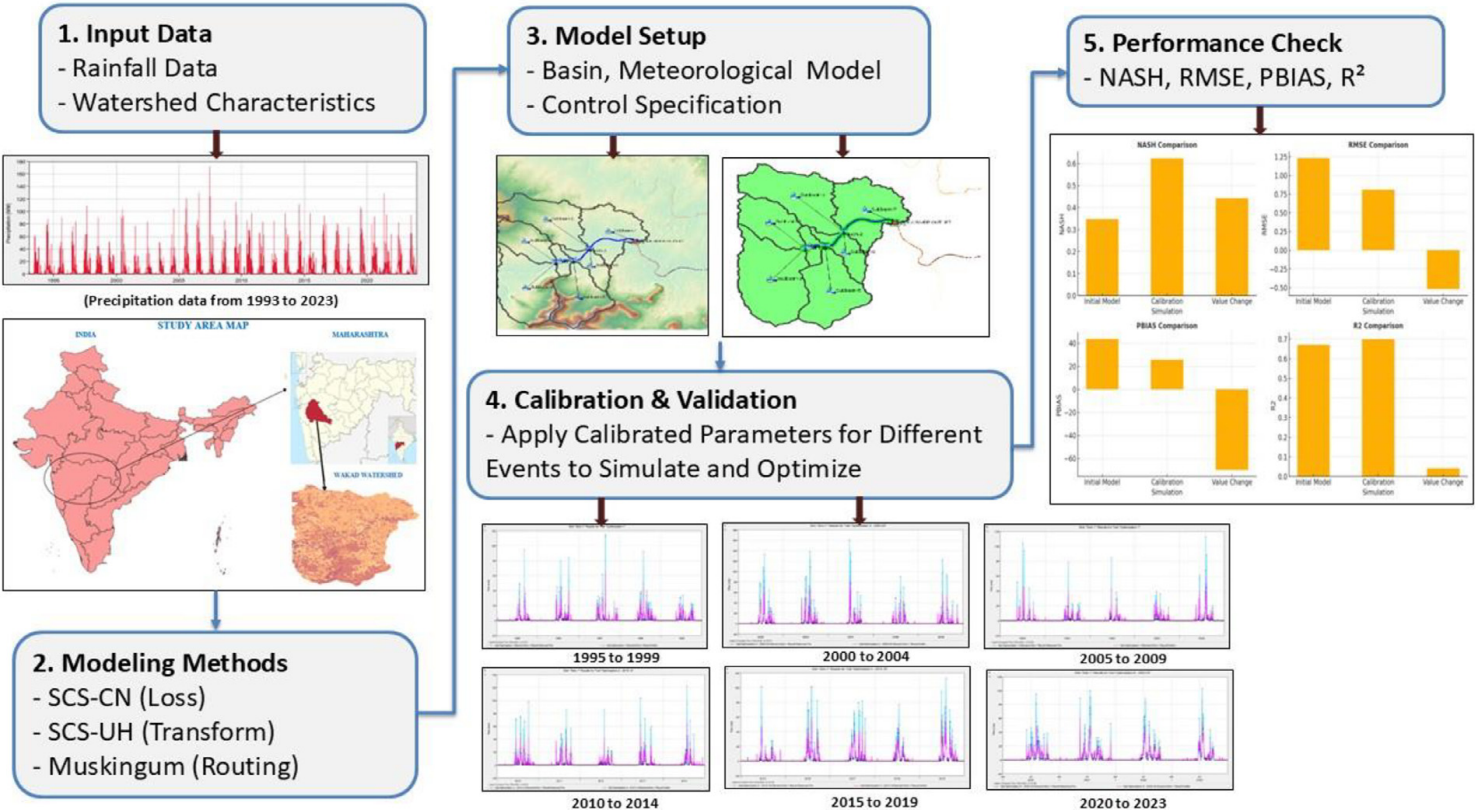
Graphical abstract.

**Fig. 2:**
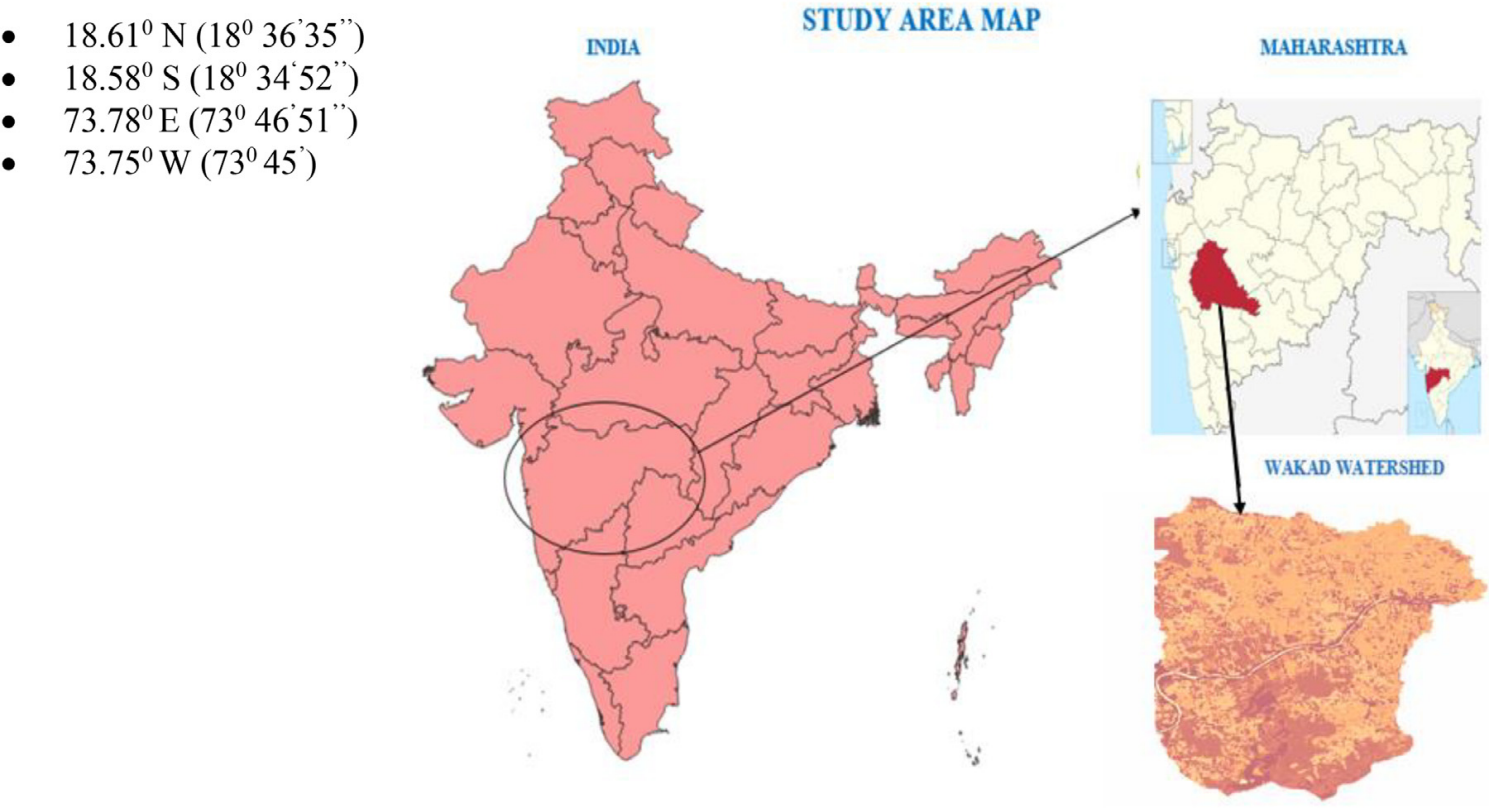
Study area.

**Fig. 3:**
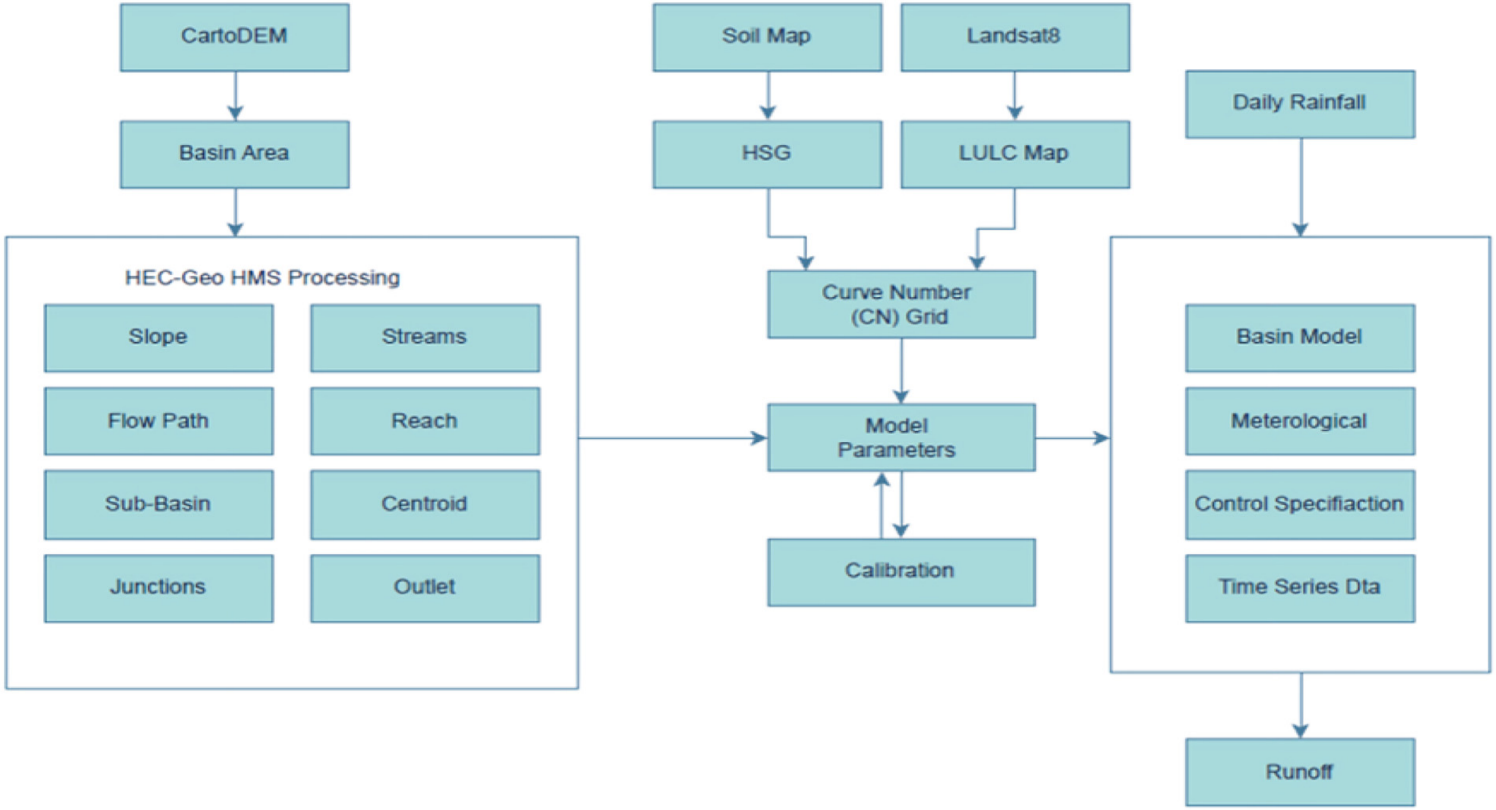
Workflow for HEC HMS.

The model set up and calibration process is crucial for accurate hydrological simulation when employing Rainfall Runoff Modelling of Urban Floods Using GIS and HEC-HMS. Model setup involves watershed delineation using a GIS software package, which delineates sub-basins, reaches, junctions, and other relevant part of the urban hydrological network pieces the rainfall-runoff model will take that into account. The hydrologic factors of the model (Curve Number (CN), time of concentration, storage values, etc.) are connected to the above-mentioned model parts either as whole units or by using data on land use, soil type, and imperviousness. The HEC-HMS modelling program is then set up to show how rainfall and flow work by showing the right loss, transform, and transport processes [16]. The model is then calibrated using both human tuning and automated optimisation tools to make the difference between the generated and actual hydrographs as small as possible. During the testing phase, past data on rainfall and flow is used to make sure that the changes in the hydrograph are real-time and can be used to judge how representative the hydrology is. Statistical measures like the Nash-Sutcliffe Efficiency Index (NSE), Root Mean Square Error (RMSE), Percent Bias (PBIAS) and R^2^ are used to test the model's performance. They show that the adjusted model is both accurate and able to predict floods Fig. 1, Fig. 2, Fig. 3.

**Fig. 4:**
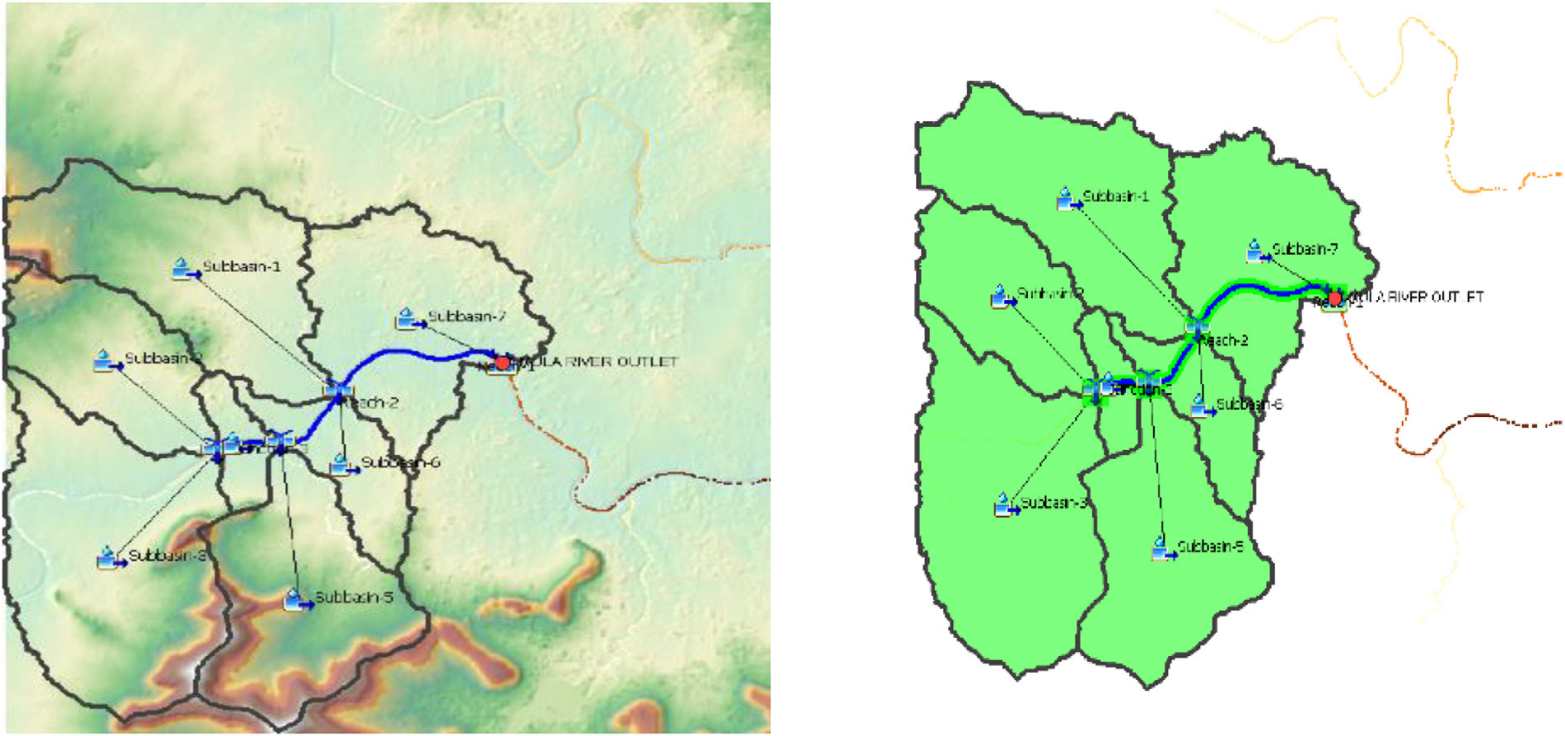
Basin model wakad watershed.

### Data acquisition

#### Digital elevation model (DEM)

**Fig. 5:**
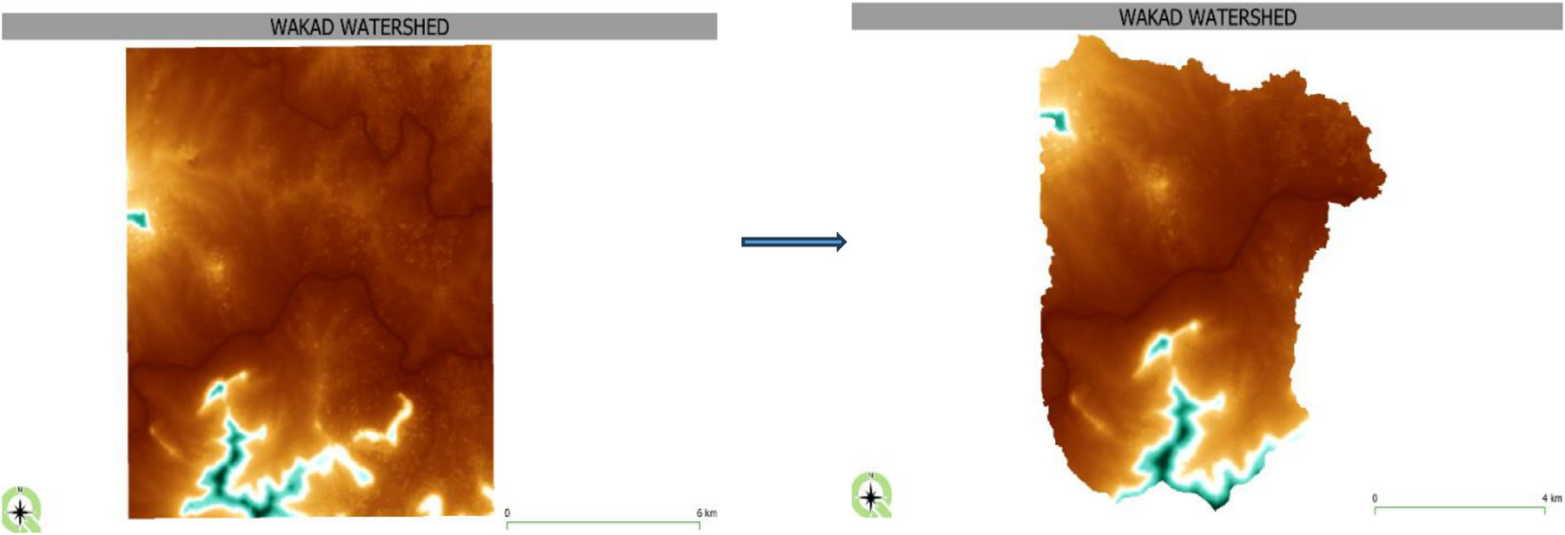
7 m resolution digital elevation model (DEM).

The analysis shows that the accurate application of secondary data provides a key role in obtaining optimal results in hydrological modelling with the help of HEC-HMS. This secondary data includes different spatial data in the form of sources of basin, the river system, sub-basin, discharge data, and rainfall stations data. The first and most grounded data is a 7 m DEM, which creates precise horizontal and vertical profiles of the earth’s terrain, important in gauging water flow and a watershed. Fig. 5 schematically shows the DEM used for this study and demonstrates its importance [17].

#### Precipitation data

**Fig. 6:**
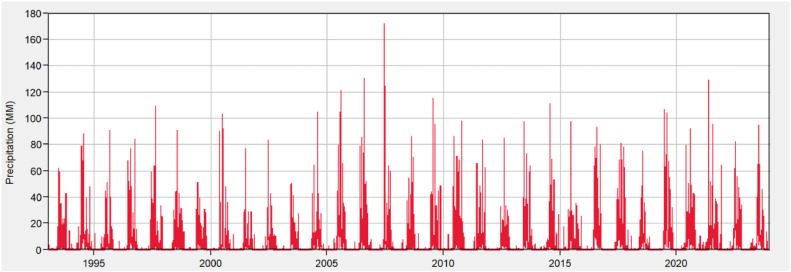
1993 To 2023 precipitation hyetograph.

Fig. 6 presents a precipitation hyetograph covering the years from 1993 to 2023 and shows the variation in rainfall over that period. The x-axis of the graph represents the years from, 1993 to 2023, while the y-axis shows precipitation in millimetres (mm). The red spikes demonstrate the different times of high rainfall, with different years demonstrating more defining peaks - demonstrating higher intensity rainfall events than others. Using the graph leads to analysing rainfall trends and identifying not just the frequency of rainfall events but also heavy rainfall events over the 30-year period.

### Parameters used in the HEC-HMS model

This paper gives an outline of the main factors used in the HEC-HMS model to simulate rainfall and flow. It talks about the different types of parts that are used, like sub-basins and reaches. Simple Surface was used to describe surface loss for the sub-basin component. It had parts for Initial Storage and Max Storage. The SCS Curve Number (CN) method is also used to describe loss [18]. This method includes the Initial Abstraction, the Curve Number, and the Impervious areas. This shows how flow is created in the sub-basin. The transform method used the SCS Unit Hydrograph (UH) method and the Time Lag method to show how the sub-basin's water levels changed when it rained. For the reach part, we used Muskingum to show how water flows through the reach using K and X. The K number is a measure of how long it takes for water to move through the reach. The above explains how the settings in HEC-HMS let it model the hydrologic reaction to a design storm and correctly guess when floods will happen based on data from the game's watershed.

### HEC-HMS and surface method

The surface method in a sub-basin models the land surface's capacity to temporarily store rainfall within the watershed. When precipitation falls on permeable areas, such as fields or open soil, it can infiltrate into the ground. However, if rain lands on impervious surfaces like roads or parking lots, no infiltration occurs, and the storage capacity is effectively zero. Surface runoff is generated when the precipitation rate exceeds the infiltration rate. The parameter called Initial Storage represents the proportion of surface storage present at the beginning of the simulation.

### HEC-HMS and soil conservation service curve number (SCS - CN)

**Fig. 7:**
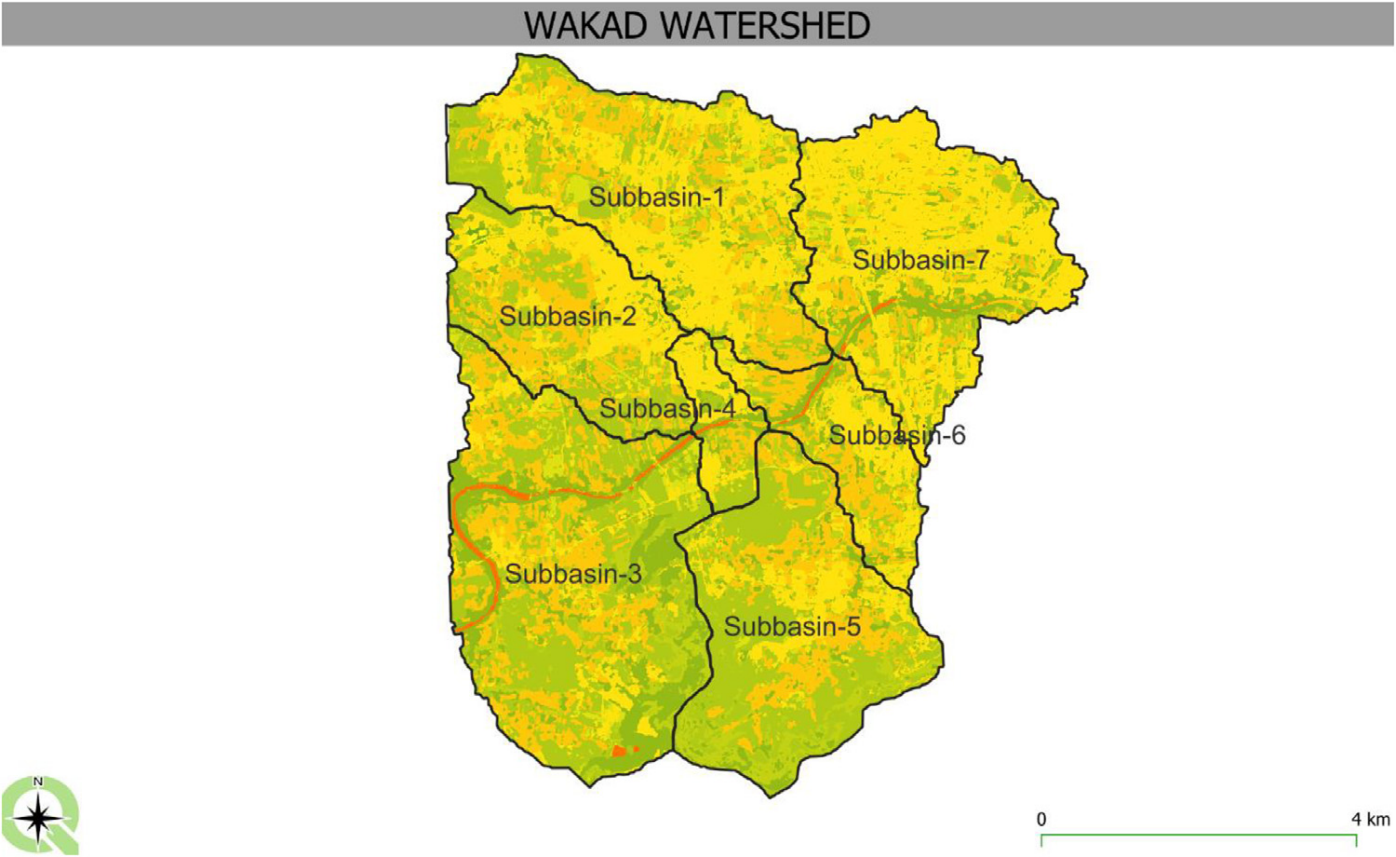
Subbasin Cn number grid.

The Fig. 7 above displays the watershed of Wakad with the Subbasin CN (Curve Number) grid. The watershed area is divided into seven sub-basins, labeled Subbasin-1 through Subbasin-7, each representing a different hydrological region with distinct characteristics [19]. The sub-basins are likely used for modeling rainfall-runoff processes, with each having a unique CN value to represent its land use, soil type, and hydrological properties. The grid illustrates how these sub-basins are spatially distributed across the Wakad watershed area.

In this study's hydrological modelling, the SCS-CN approach is an appropriate way for calculating runoff based on the type of dirt, how the land is used, and the water conditions. The method uses curve numbers to measure entry and storm flow and to guess the likely maximum retention and beginning abstraction [20]. To guess the amount of direct flow in a certain area, the Soil Conservation Service Curve Number (SCS-CN) method can be used. This is a well-known hydrology model. Based on the water balance, it provides a more realistic way to measure runoff from a specific rainfall event. According to the technique you mentioned, the main assumptions and equations need a good grasp of how runoff is calculated based on rainfall, initial abstraction, and the watershed's retention characteristics. Now, let's look at the main aspects of the streptococcal approach [21].

***1. Water Balance Equation*** (Eq. (1))

The fundamental water balance equation, as given in the problem, is:

(1) $$P=I_{a}+F+Q$$

Where:

• P = Total rainfall (in inches or mm)
• I_a_ = Initial abstraction (inches or mm), representing losses due to interception, infiltration before runoff begins, etc.
• F = Actual infiltration (inches or mm)
• Q = Direct runoff (inches or mm)

This equation essentially states that the total rainfall P is partitioned into three components: initial abstraction (I_a_), infiltration (F), and runoff (Q).

***2. Proportional Equality Hypothesis*** (Eq. (2))

The second hypothesis formulated a hydrology link between actual infiltration (F) and runoff (Q) in a watershed, adjusted for the maximum retention capability (as a fraction of rainfall), S and the aggregate rainfall (P). According to this method, true infiltration is calculated by the ratio of observed runoff in relation to the maximal water storage which depends on the degree of runoff concentration after eliminating the initial abstraction which does not contribute to runoff but is lost by other ways, for example, by interception, or stored in the form of soil moisture [22].

(2) Q/P−Ia=F/S

This principle is widely used in Hydrological modeling, for instance SCS-CN or Soil Conservation Service Curve Number, for determination of runoff and infiltration in the watershed.

***3. The First Hypothesis of Abstraction*** (Eq. (3))

The first hypothesis of abstraction in this scheme formulates quantitative interaction between the initial abstraction (I _{a}) and the maximum number of George Orwell’s works to retain (S) through a proportional factor λ. This principle is mathematically depicted as

(3) Ia=λS

Here, λ is used as a scaling factor; typically set at 0.2 as this has been found to produces good results. This means that, the first abstraction that precedes the retention process, indicates the extent of loss of capacity before it is retained, is an indicator of the maximum retention capacity [23]. The constant λ allows for the definition of how much of the total capacity is being used before retention begins to become effective and the concept fits well into the hydrological modelling world broken down into compartments.

***4. Runoff Equation*** (Eq. (4))

When combining the water balance equation (Eq. (1)) and the proportional equality (Eq. (2)), the result is an expression for runoff Q:

(4) $$Q=\left(P-I_{a}\right)2/P-I_{a}+S$$

This formula holds true when P ≥ I_a_ i.e., when rainfall exceeds the initial abstraction and runoff begins.

***5. Modified Equation for λ=0.***2 (Eq. (5))

Assuming that the initial abstraction I_a_ is 20 % of the maximum retention (i.e., λ=0.2), the runoff formula becomes:

(5) Q=(P−0.2S)2/P+0.8S

This is the standard form of the SCS-CN runoff equation and is typically used in hydrological modelling.

***6. Relationship Between Curve Number (CN) and S*** (Eq. (6))

The SCS-CN method also connects the Curve Number (CN) to the maximum retention S. The relationship is given by:

(6) S=(CN/1000)−10

Where:

• The SCS curve number, or CN, is a dimensionless metric that is influenced by the watershed's hydrological state, soil composition, and land use.
• S is the maximum potential retention in inches.

A lower CN number indicates more permeable soils and less runoff, whereas a higher CN value indicates less permeable soils and more runoff. The Curve Number falls between 0 and 100.

***7. Time of Concentration and Lag Time*** (Eq. (7))

The SCS method also provides a way to estimate time of concentration (T_c_), which is the duration of water flow from the watershed's furthest point to its outlet. The equation for Tc is:

(7) $$T_{c}=l0.8\left(S+1\right)0.7/1140\;Y0.5$$

Where, T_c_ is the time of concentration (in hours), The longest flow path's flow length, expressed in feet, is l, Y represents the watershed's average land slope (in percentage), S is the maximum retention possible (inches) and CN is the Curve Number.

(8) $$T_{\text{lag}}=0.6\cdot T_{c}$$

Time of concentration is important for understanding how long it takes for the runoff to reach the watershed outlet. This, in turn, helps in estimating the lag time (T_{lag}) by multiplying T_c_ by a factor of 0.6.

### HEC – HMS transform method

The transform method within each sub-basin is responsible for converting effective rainfall into a corresponding runoff hydrograph. This transformation simulates how rainfall is translated into flow over time, reflecting the dynamic response of the watershed. In this study, the Soil Conservation Service Unit Hydrograph (SCS-UH) method is employed as the transform method. The SCS-UH technique is widely used for its simplicity and reliability in representing watershed response, particularly in small to medium-sized catchments. A key parameter in the SCS-UH method is the lag time, which is defined as the time difference between the centroid of the effective rainfall and the peak of the resulting runoff hydrograph. Lag time essentially represents the delay in runoff response and is influenced by watershed characteristics such as slope, land use, soil type, and drainage network. For this analysis, the lag time for each sub-basin is computed individually based on its physical attributes.

### Muskingum routing method

In the HEC-HMS hydrologic modelling framework, flow routing simulates the movement of water through channels or reaches from upstream to downstream. This process is essential to determine how inflow hydrographs are transformed as they travel through river reaches, especially in flood modelling and timing of peak flows. In this study, the Muskingum method is employed for routing flow through channels within the watershed. The Muskingum method is based on the continuity equation and assumes that the storage in a river reach is a function of both inflow and outflow. The total storage S in the reach is expressed as:

S=K[xI+(1−x)Q]

Where:

• S = Storage in the reach (m³ or equivalent)
• I = Inflow rate (m³/s)
• Q = Outflow rate (m³/s)
• K = Storage time constant (travel time through the reach)
• x = Weighting factor (ranges from 0 to 0.5), representing the influence of inflow on storage

Each reach within the Wakad Watershed is assigned Muskingum routing parameters based on its length, slope, and flow characteristics. These parameters help simulate attenuation of peak flows, delays in hydrograph timing, and volume conservation as water moves downstream.

## Calibration and validation HEC HMS

The study on urban flood modelling through GIS and HEC-HMS integrates advanced precipitation and runoff modelling methods to understand flood dynamics in the Wakad watershed over three decades (1993–2023). The methodology utilizes specified hyetographs, the SCS Curve Number (CN) method to figure out waste amount and the Muskingum method to figure out how to get from one place to another. The SCS Unit Hydrograph (SCH UH) method is used to change the data and simulate direct flow. This method shows how complicated urban hydrology is. The model was calibrated and validated from 1995 to 2023, and it worked to show that values from the Nash-Sutcliffe Efficiency (NASH), RMSE, PBIAS and R^2^ statistics were used to show where the actual and simulated hydrographs were most similar. During testing, the model not only worked well, but the NASH went up and the RMSE went down. This shows that using the right parameters and assumptions and mixing different rainy periods can lead to accurate model forecasts of runoff. The model is important for good and calibrated predictions of flood hazards for identifying urban flood management strategies and developing safer and resilient urban areas within the built environment.

### Key performance metrics in calibration and validation

The following metrics are commonly used to assess model performance during calibration and validation:

• **Nash-Sutcliffe Efficiency (NASH)**:
  ○ Formula:
    (9) $$NASH=1-\frac{\sum_{t=1}^{N}{\left(Q_{obs,t}-Q_{sim,t}\right)}^{2}}{\sum_{t=1}^{N}{\left(Q_{obs,t}-{\overset{‾}{Q}}_{obs}\right)}^{2}}$$
  ○ Where $Q_{obs,t}$ is the observed flow, $Q_{sim,t}$ is the simulated flow, and ${\overset{‾}{Q}}_{obs}$ is the mean observed flow.
  ○ Values closer to 1 indicate a better fit.
• **Root Mean Square Error (RMSE)**:
  ○ Formula:
    (10) $$RMSE=\sqrt{\frac{1}{N}\sum_{t=1}^{N}{\left(Q_{obs,t}-Q_{sim,t}\right)}^{2}}$$
  ○ Lower values indicate better model performance.
• **Percent Bias (PBIAS)**:
  ○ Formula:
    (11) $$PBIAS=\frac{\sum_{t=1}^{N}\left(Q_{sim,t}-Q_{obs,t}\right)}{\sum_{t=1}^{N}Q_{obs,t}}\times \;100$$
  ○ PBIAS values close to 0 indicate a good match between observed and simulated values.
• **Coefficient of Determination (R²)**:
  ○ Formula:
    (12) $$R^{2}=\frac{{\left(\sum_{t=1}^{N}\left(Q_{obs,t}-{\overset{‾}{Q}}_{obs}\right)\left(Q_{sim,t}-{\overset{‾}{Q}}_{sim}\right)\right)}^{2}}{\sum_{t=1}^{N}{\left(Q_{obs,t}-{\overset{‾}{Q}}_{obs}\right)}^{2}\sum_{t=1}^{N}{\left(Q_{sim,t}-{\overset{‾}{Q}}_{sim}\right)}^{2}}$$
  ○ R² values closer to 1 indicate a higher correlation between observed and simulated data.

These metrics give quantitative values that are used to assess the accuracy of the HEC-HMS model when calibrated and validated. All model output is then adjusted accordingly based on the final results of each of the metrics in order for the model to accurately represent the watershed behavior under many sampling rainfall conditions.

**Fig. 8:**
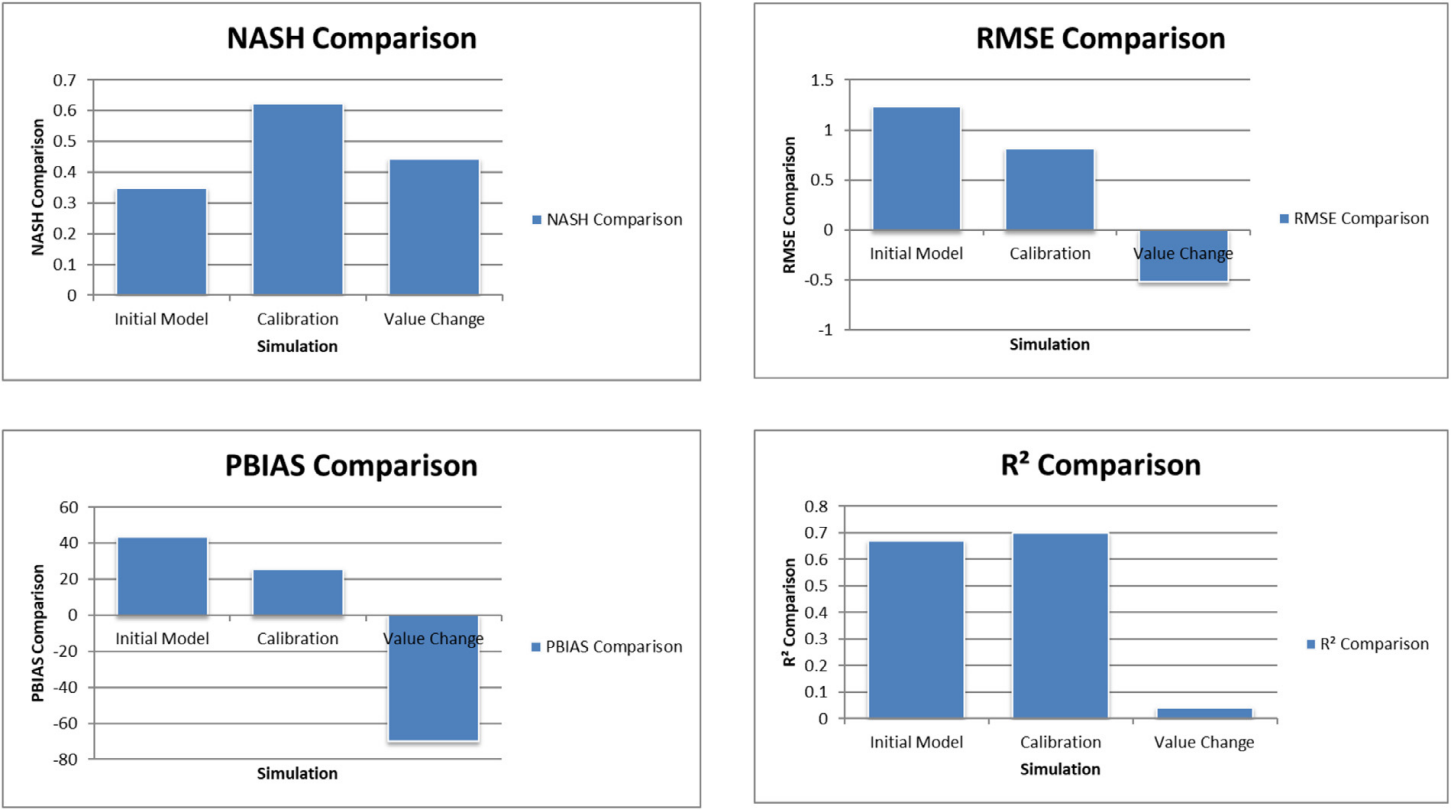
Graphical representation of HEC HMS model performance metrics.

**Fig. 9:**
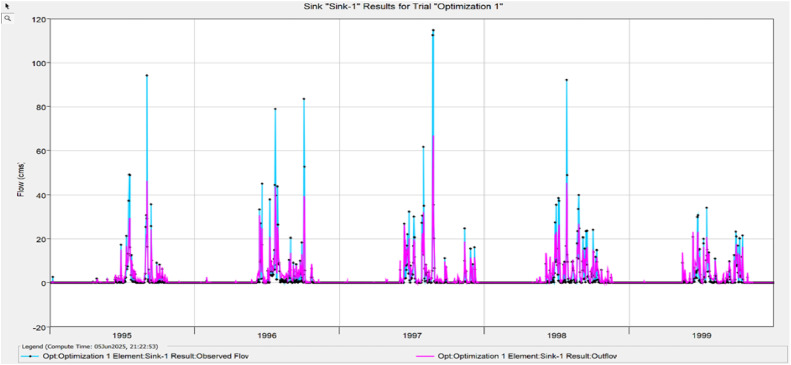
Simulation and optimisation from 1995 to 1999.

**Fig. 10:**
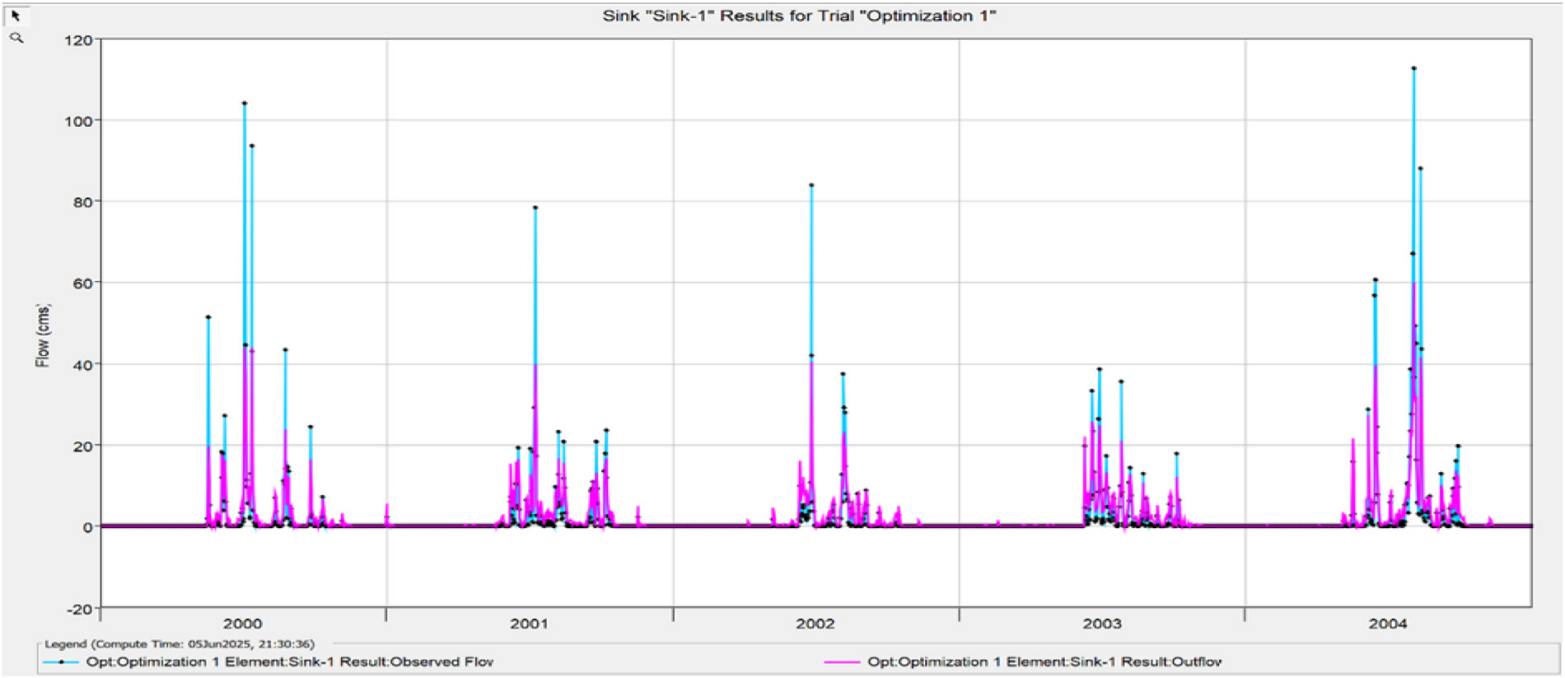
Simulation and optimisation from 2000 to 2004.

**Fig. 11:**
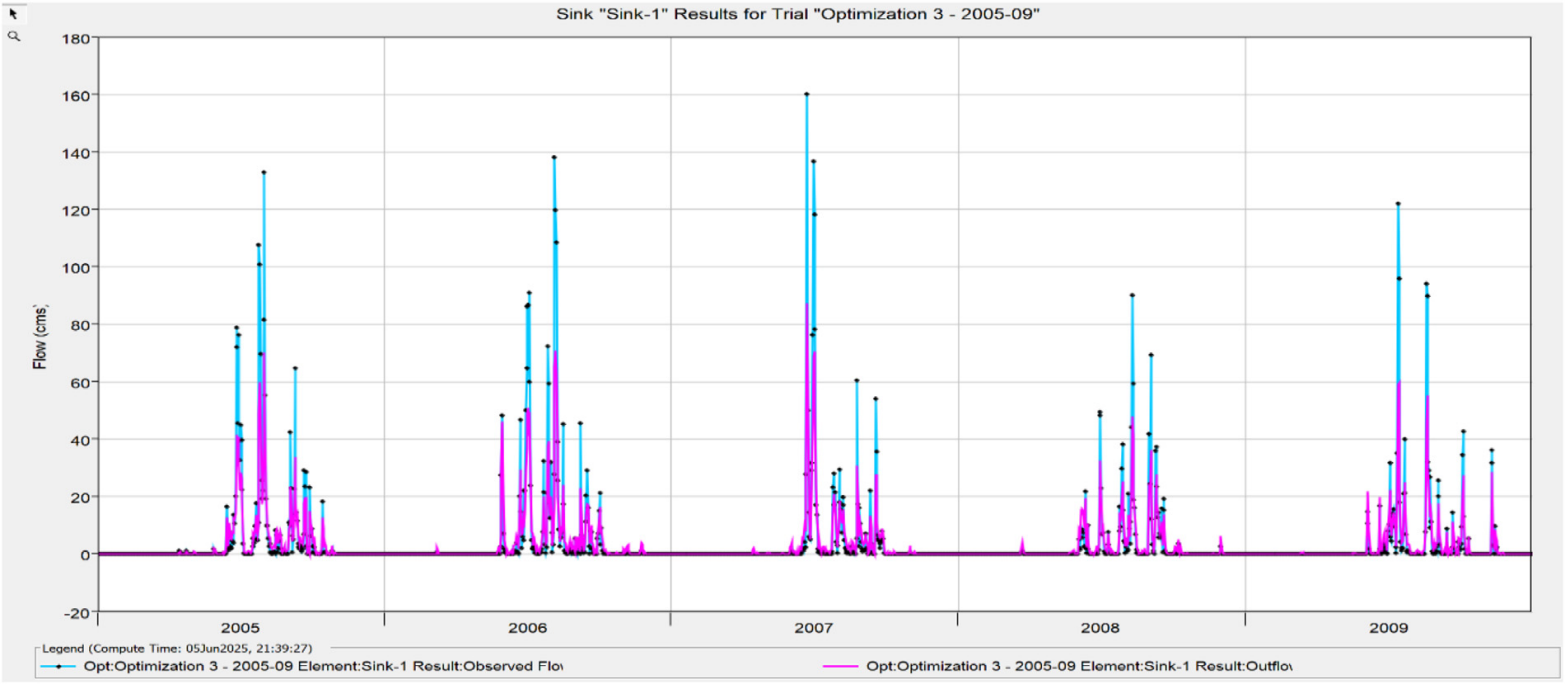
Simulation and optimisation from 2005 to 2009.

**Fig. 12:**
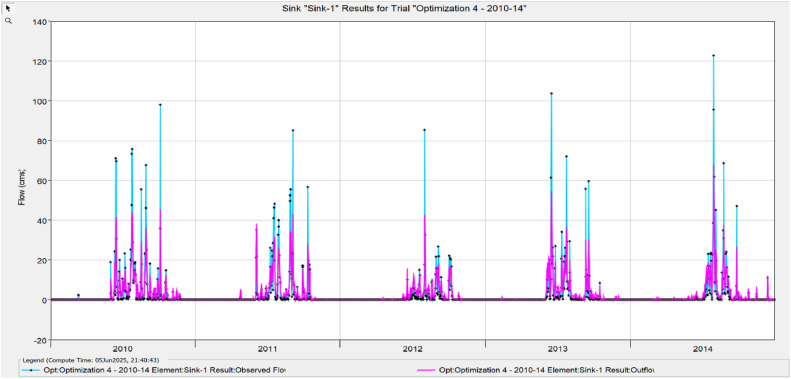
Simulation and optimisation from 2010 To 2011.

**Fig. 13:**
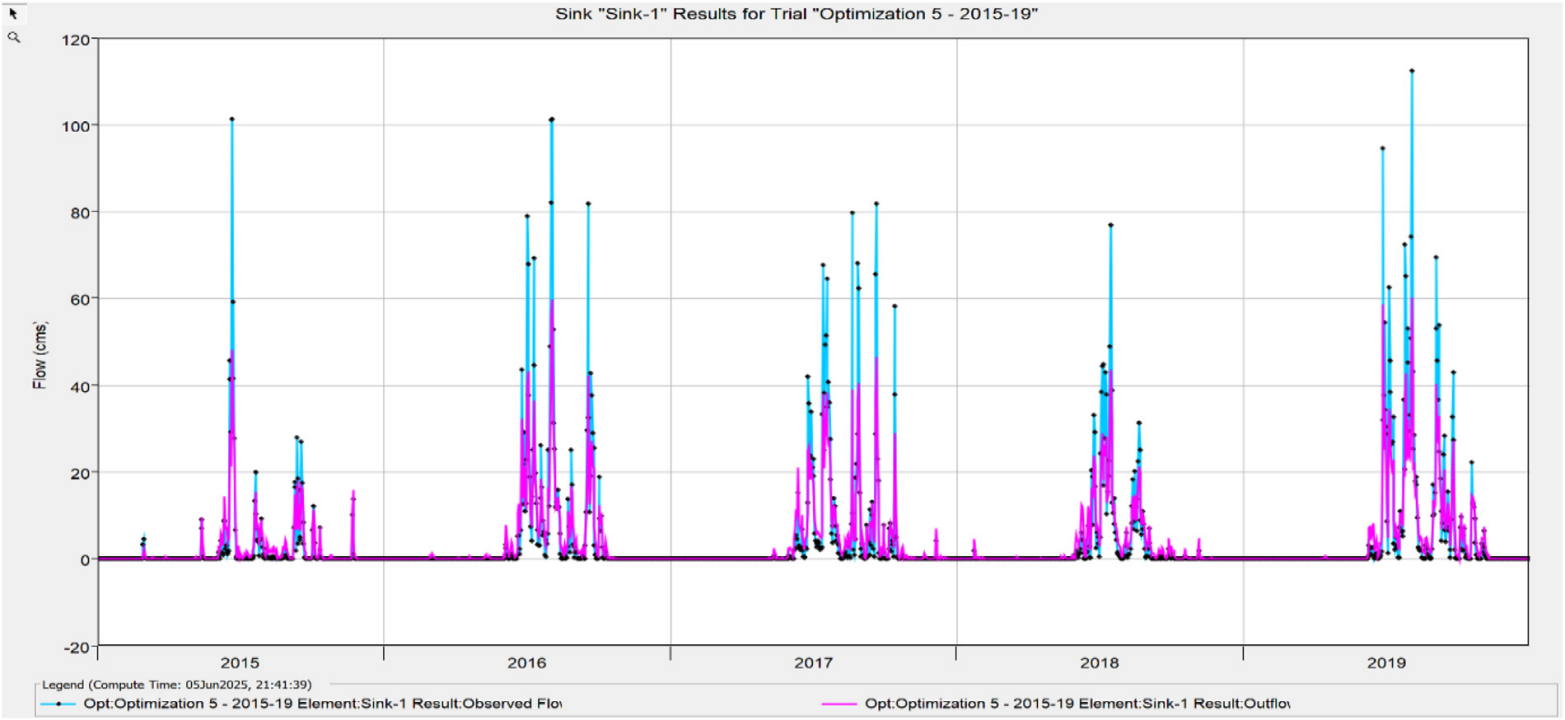
Simulation and optimisation from 2015 to 2019.

**Fig. 14:**
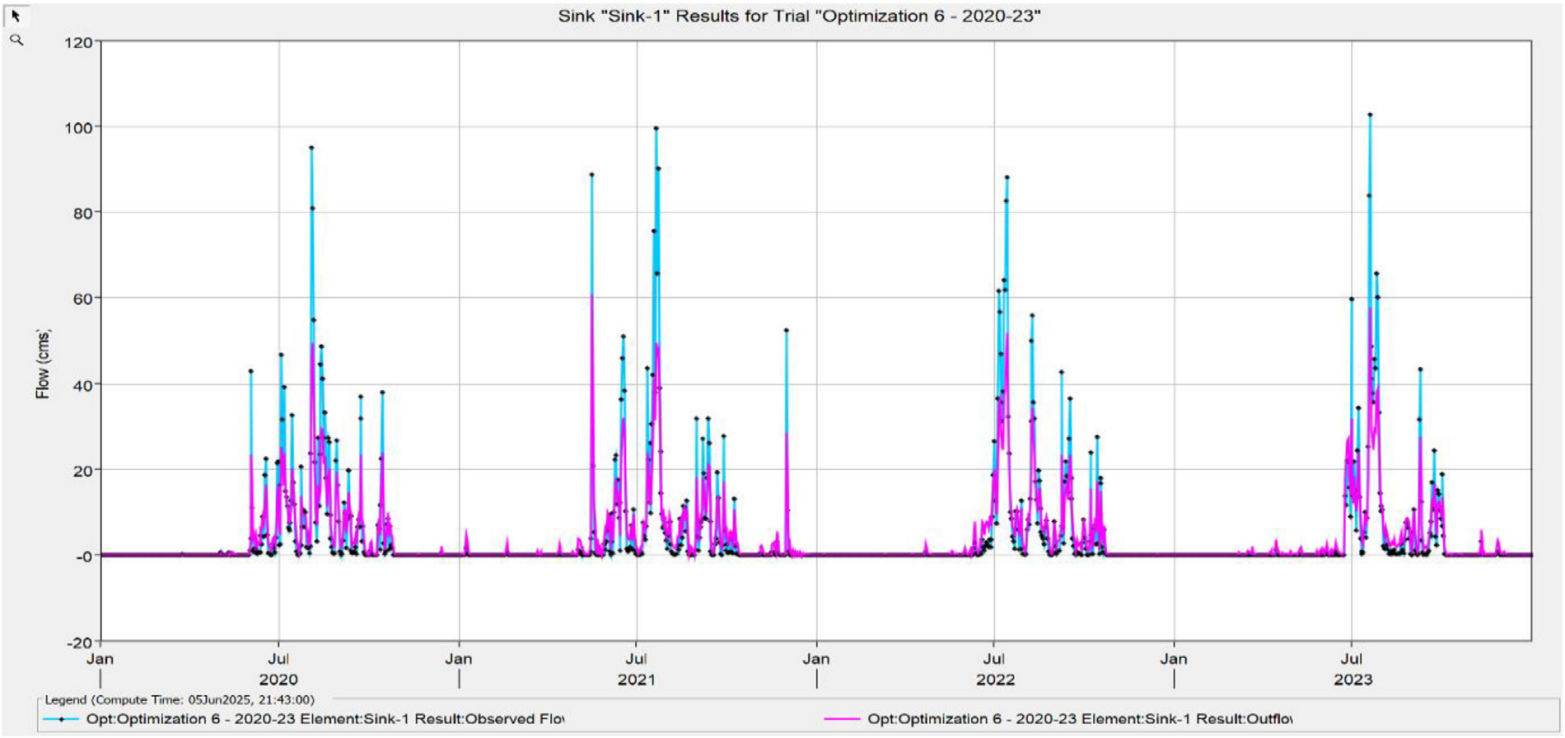
Simulation and optimisation from 2020 to 2023.

The Model Performance Metrics Table 5 shows the evaluation of the model at different stages: initial and calibration. For the initial model, the Nash-Sutcliffe Efficiency (NASH) is 0.348, indicating low model performance, with a RMSE of 1.235, which reflects a higher error. The PBIAS is 43.69 %, suggesting an overestimation of runoff, and R2 is 0.67, indicating moderate correlation between observed and simulated data. During calibration, performance improves significantly, with NASH increasing to 0.624, RMSE decreasing to 0.813, PBIAS reducing to 25.70 %, and R2 rising to 0.699. The value change percentages highlight the improvement in the model's accuracy Fig. 8, Fig. 9, Fig. 10, Fig. 11, Fig. 12, Fig. 13, Fig. 14.

**Table 5:** Model performance metrics table.

SIMULATION	NASH	RMSE	PBIAS	R^2^
Initial Model	0.348	1.235	43.69 %	0.67
Calibration	0.624	0.813	25.70 %	0.699
Value Change	44.23 %	-51.90 %	-70 %	4.14 %

### Hydrograph comparison between simulated and observed flow for Wakad watershed

The HEC-HMS model for the Wakad Watershed was calibrated and proven to work by comparing hydrographs of synthetic and real-world flow data. The simulations were accurate from 1995 to 1999, 2000 to 2004, 2005 to 2009, 2010 to 2011, 2015 to 2019, and 2020 to 2023. Each time period showed a good match between the calculated and actual data. This proved that the model is accurate for simulating urban floods and can make the best forecasts for the future.

## Recommendations and policies

The research suggests the coupling of GIS and HEC-HMS modeling is a feasible and reliable possibility for urban flood management techniques of rapidly urbanizing locations (i.e, Wakad Watershed) in Pune. The validation results based on simulations, weather events, 30 years of precipitation data, and DEM approach support the application of event-based simulations to estimate peak discharge and runoff volumes. The efficiency of event-based simulations is proven to provide better estimates of urban inundation runoff volumes and durations than continuous simulations. These findings provide an assessment of the study area model to accurately reflect similar hydrological responses in nature, and better predict the potential for urban flooding risk in the future. In addition to concluding potential policy implications for best practice flood risk and sense of place urban planning management framework recommendations, which include formal land use policy restrictions that provide impermeable surface limits, a “once in 10 years” reassessment of watershed conditions and infrastructure, and continuing to develop drainage systems and infrastructure capable of accommodating increased runoff conditions with anticipated continued urban development with the onset of climate change. Overall, the integrated hydrologic modeling procedures taken can provide support for adaptive urban planning decisions using an evidence-based approach to minimize urban flooding damage, improve urban resilience to flooding in an adaptive way, sustainably manage urban water resources, and protect both property and lives from urban flooding risk where urban landscapes have been prone to flooding previously.

## Conclusion

The research on urban floods' rainfall-runoff modeling with GIS and HEC-HMS in the Wakad Watershed provides valuable knowledge about flood dynamics and hydrological processes. The HEC-HMS model's calibration and validation showed 30, years (1993–2023) accuracy levels improved significantly. The 7-meter resolution, high-resolution GIS data, and long-term precipitation data records provided excellent sources for watershed characterization. The Soil Conservation Service Curve Number (SCS-CN), the SCS Unit Hydrograph (SCS-UH), and Muskingum routing methods implemented successfully, showing that the model is a strong modeling tool to estimate runoff for urban flood management and watershed model research This study evaluated the performance of a hydrologic model developed for the Wakad Watershed using HEC-HMS, with particular emphasis on improving model accuracy through calibration. Key performance metrics; including Nash-Sutcliffe Efficiency (NASH), Root Mean Square Error (RMSE), Percent Bias (PBIAS), and Coefficient of Determination (R²) were used to assess the effectiveness of the calibration process. The model demonstrated a marked improvement post-calibration. NASH increased from 0.348 to 0.624, reflecting a 44.23 % enhancement in the agreement between observed and simulated flows. RMSE decreased significantly from 1.235 to 0.813, indicating a 51.90 % improvement in predictive accuracy. Furthermore, PBIAS was reduced from 43.69 % to 25.70 %, amounting to a 70 % reduction in bias. The R² value, although already acceptable, improved slightly from 0.67 to 0.699, suggesting a 4.14 % increase in the model's ability to explain variance in the observed data.. These improvements validate the robustness and applicability of the calibrated model for hydrologic analysis in urbanizing watersheds. The findings support the use of the HEC-HMS model as a reliable tool for simulating rainfall-runoff dynamics, and for guiding sustainable water resource management and flood mitigation planning in rapidly developing regions such as Wakad, Pune. This work underscores the importance of advanced tools like HEC-HMS and GIS for sustainable urban flood management and water resource planning, offering valuable insights for infrastructure upgrades in urbanizing regions.

## Ethical approval

The submitted work is original and not have been published elsewhere in any form or language (partially or in full), unless the new work concerns an expansion of previous work.

## Consent to participate

Informed consent was obtained from all individual participants included in the study.

## Consent to publish

The authors affirm that human research participants provided informed consent for publication of the research study to the journal.

## Funding

The authors declare that no funds, grants, or other support were received during the preparation of this manuscript.

## CRediT authorship contribution statement

**Priyanka Sharad Jawale:** Conceptualization, Resources, Data curation, Formal analysis, Writing – original draft, Writing – review & editing. **A.D. Thube:** Writing – review & editing, Conceptualization, Resources, Data curation, Formal analysis.

## Declaration of competing interest

The writers say they don't have any known personal or financial ties or financial interests that could have seemed to affect the work in this study.

## Data Availability

The data that has been used is confidential.
